# A scoping review of the association between rural medical education and rural practice location

**DOI:** 10.1186/s12960-015-0017-3

**Published:** 2015-05-06

**Authors:** Jane Farmer, Amanda Kenny, Carol McKinstry, Richard D Huysmans

**Affiliations:** College of Science, Health and Engineering, La Trobe University, Melbourne, VIC 3086 Australia; La Trobe Rural Health School, La Trobe University, Bendigo, VIC 3550 Australia; Department of Allied Health, La Trobe Rural Health School, La Trobe University, Bendigo, VIC 3550 Australia; Raven Consulting Group, Victoria, Australia

**Keywords:** Rural, Medical, Doctors, Graduate, Education

## Abstract

**Background:**

Inequitable distribution of the medical workforce is an international problem that undermines universal access to healthcare. Governments in many countries have invested in rural-focused medical education programs to increase the supply of rural doctors.

**Methods:**

Using a structured five-step approach, a scoping review was conducted to map the existing evidence on the relationship between professional entry-level, pre-vocational medical education delivered in rural settings and rural workforce outcomes. Key search terms were developed, with database searches yielding 37 relevant articles. During data charting, a set of types of studies emerged, and we developed a typology to assist with article sorting and information structuring.

**Results:**

Medical students attending a rural campus or spending time in a rural area are more likely to practise in non-metropolitan areas upon graduation than students studying at a city campus. In many cases, these positive findings could be confounded by students having a rural origin or being predisposed to want rural work. There is some evidence to suggest that the longer a person spends time as a medical student in a rural area, the more likely they are to work rurally following graduation. Overall, the articles located had limitations related to small sample size, inconsistent definition of rurality and lack of attention to controlling for variables that might influence rural practice decision, for example, rural background. Comparative data were lacking, and most studies were conducted by staff from the medical schools that were the focus of the research. There was no consideration given in any study found to the cost-effectiveness of entry-level medical education delivered in rural settings versus other ways of producing rural practitioners.

**Conclusions:**

Given limitations, available evidence suggests that medical education in a rural location does increase the number of medical graduates that will work in a rural place. There are indications of a gradient effect where increased rural practice exposure during medical education leads to more rurally located graduates; however, robust studies are needed to verify this finding. Given the significant funding being directed to universities to increase graduates that will work rurally, appropriate future research is recommended.

## Introduction

In this article, we review what is known about an association between professional entry-level, pre-vocational medical education in rural areas and rural medical practice location following graduation. We address two research questions: does medical education in a rural place produce rural doctors? And, is there a gradient relationship between time spent in rural-based medical education and likelihood of working in rural practice? For the purposes of this review, our interest was rural practice following graduation from a medical school, so we did not include the impact of postgraduate study. We acknowledge that there exists evidence indicating diverse factors associated with practising rurally. In this article, our specific interest is in studies that explored associations between rural medical education and rural practice location of doctors.

Internationally, there is significant funding invested in initiatives to increase the numbers of medical graduates choosing rural practice. While there is a body of evidence that suggests rural upbringing as a predictor of rural medical practice [1-3], less attention has been given to specifically considering the effects of place of education.

Especially in a constrained financial environment, funding decisions about medical student programs should be based on sound evidence. The purpose of our review is to map the evidence and summarise what is known about relationships between entry-level, pre-vocational medical education delivered in rural places and rural work outcomes. Rural as a spatial categorisation is a contested concept and, internationally, is subject to multiple definitions [4]. For the purposes of our work, we have included studies where the authors state their interest is rural and define or describe it. We allowed the use of synonyms—remote and regional.

### Background

Inequitable distribution of the medical workforce is an international problem that undermines universal access to health services [5,6]. Although direct evidence of causation between medical professional presence and better health is illusive, studies have associated higher physician–population ratios with higher overall state health rankings in the USA [7] and better self-reported general health in Canada [8]. Places with poorer health and few medical practitioners tend to coincide. Given that service accessibility is an established social determinant of health [9], addressing medical workforce maldistribution is important.

Evidence shows that physician assistants, nurse practitioners and community health workers can substitute for high proportions of medical practitioner competencies [10], but medical doctors have the widest scope of practice [11] and are integral to healthcare provision in any context. Access to sufficient medical care, as a basic human right to achieve adequate health and well-being, has been enshrined in United Nations policy since 1948 [12].

Inequities in doctor distribution are most significant when rural–urban comparisons are made [5]. Per 100 000 people, Canada has approximately 100 physicians in rural areas, compared with 150 for urban [13]. Australian figures are 98.5 per 100 000 in very remote areas, compared with 266 for state capitals [14]. A Japanese study showed 185 doctors per 100 000 in rural, compared with 272.5 for urban prefectures [15].

The rural general medical practitioner (GP) is skilled to deal with diverse encounters and contexts, clinical procedures, after-hours work, emergencies, complex care, public health issues and Indigenous peoples’ needs [16]. Practising medicine in rural hospitals involves the following:

*…*[a] *broad generalist set of skills, knowledge and attitudes…practiced at a distance from comprehensive specialist medical and surgical services and investigations.* [17]

### Rural medical staff

Historically, attracting rural medical staff to improve health and social conditions is a government concern. For example, the 1912 Dewar Report established the Scottish Highlands and Islands Medical Service, precursor to the UK National Health Service (NHS), to address poor rural health outcomes and a dearth of rural doctors [18].

Attracting medical graduates to work in rural areas is challenging, with recent United States (USA) research indicating only 3% of medical school entrants planned to practise in rural areas [19], compared with 19.3% of the USA population living rurally [20]. The inability of governments to ensure sufficient entrants to rural medical practice has resulted in numerous initiatives aimed at increasing rural medical graduate supply—including bonded student places, clinical schools, workforce agencies, work incentives and medical undergraduate degree programs or pathways focused specifically on rural practice [21]. In many countries, there has been major investment in such strategies, but the World Health Organisation (WHO) [6] notes a lack of robust evaluations and limited evidence for their effectiveness in increasing the overall supply of rural doctors.

### Australia as an example: the expenditure–evidence gap

To grow rural doctor supply from domestic students, a strong focus of Australian investment has been the funding of city-based medical schools to develop rural components in degree programs. In the Australian context, initial medical education typically lasts from 4 to 6 years. Students enter undergraduate programs direct from secondary school, or graduate entry programs, where students are admitted on the basis of completing an earlier undergraduate degree. The rural component of Australian programs tends to be delivered largely through rural clinical schools. The University Departments of Rural Health and Rural Clinical School (UDRH/RCS) programme began in 1994 [21]. The Australian Rural Clinical Training Scheme (RCTS) targets include the following: 25% of all medical students to have at least 1 year of rural clinical training by graduation, 25% of government-supported medical students to be recruited from a rural background, and all government-supported medical students to have at least 4 weeks of rural placement [21]. Supporting a ‘trickle-down’ strategy [22], government-supported medical places have substantially increased, and from 2014, Australia will have tripled overall medical graduate numbers since 2001 (to around 3 800 a year) [21].

Noting proliferation of initiatives to encourage rural working, an Australian government review [21] identified the need for a better evidence base on rural medical education and systematic monitoring of rural career choices. The report is consistent with academic reviews that identified the need for ‘comprehensive, methodologically rigorous longitudinal studies’ of outcomes from rural medical investment [23].

### New rural medical schools

In an era of fiscal constraint, Australian universities have attracted funding for new medical programmes built on a case of addressing rural disadvantage. ‘Rural’ medical schools have been developed in Geelong and Wollongong, although both are categorised as ‘major cities’ using the Australian Standard Geographical Classification [24]. James Cook University medical school in Townsville, Queensland, and Flinders University Northern Territory Medical Program, based in Darwin, Northern Territory, are recently developed medical programmes located in ‘outer regional areas’.

Establishing medical programmes in rural places is not a purely Australian phenomenon. The Northern Ontario School of Medicine was established in 2005 as a ‘rural, distributed community-based medical school’ recruiting students from Canadian northern, rural, remote, Aboriginal or Francophone backgrounds [25]. Exeter and Plymouth medical school (now two separate schools) was established in 2000 to provide medical education for the peripheral English southwest area.

### Medical programmes in rural locations

An evidence base exists that identifies predictors for medical graduates choosing to practise rurally. Rural background and duration of time spent living in a rural place, prior to medical education, have been consistently described as strong predictors [26-28].

Considering associations between experience of living rurally and rural practice following graduation, Farmer et al. [29] use Bourdieu’s notion of habitus (expectations and understanding, based on experience of living in an environment with certain cultural features, that shape one’s sense of the social world) to explain why extended rural experience predicts comfort with rural work and living [30]. Researchers have confirmed the importance of rural ‘acculturation’ in practice choices [31].

Building on evidence of associations between time residing rurally and eventual work outcomes, some argue that the key to increasing rural practitioners is to develop medical schools where all education is delivered in a rural place. The success of such programmes, for example, that of James Cook University, where 59% of 2005–2011 graduates primarily worked outside of major cities postgraduation [32], has fuelled interest in wholly rural medical education.

### A polarised debate

There is vigorous debate about the need for new medical programmes located wholly in rural locations. Proponents argue rural location is a key feature for addressing workforce disparity, while metropolitan-based medical schools argue their programmes with rural components are effective and increasing medical student places is unnecessary [33]. The issue is complex. Overall increases in medical student numbers, especially if exposed to rural experience, could lead to more rural doctors. However, actual effects of increasing graduate output on workforce distribution are unknown, partly due to the novelty of many initiatives.

With increasing higher education marketisation, the debate intensifies. Medical schools are attractive for universities in any country [34]. Universities are attracted by high payments for training medical students (for example, in 2014, the Australian government paid universities $21,273 per medical student place, compared with $13,163 for nursing [35]). Internationally, universities are attracted to medical schools as their biomedical research produces high-research income, and international university rankings tend to be weighted in favour of biomedical research outputs [36]. Across all countries, there is high demand for medical student places from a growing middle class [37].

In an environment characterised by these debates, having good, independent, evidence to inform funding decisions about medical school places would be useful. While the weight of evidence shows an association between rural upbringing and practising rurally [26-28], the impact of professional entry medical education in rural places on increased rural workforce capacity appears less clear. It is this opaqueness that prompted our review.

## Methods

A scoping review was undertaken to identify what was known about the impact of professional entry medical education in rural places on rural medical practice location following graduation. Scoping reviews are an effective, robust approach to map, collate and summarise literature [38]. This type of review is useful to map research areas, as unlike systematic reviews, all study designs can be included. The focus is on what knowledge exists, rather than on research quality [39]. We used Arksey and O’Malley’s [39] five-stage framework for scoping reviews: identify the research question; identify relevant studies; study selection; chart the data; and collate, summarise and report results.

### Defining the research question

Consistent with Arksey and O’Malley’s [39] recommendations, a broad question and key terms were used to ‘generate breadth of coverage’. Our aim was to maximise the range of literature captured, so the question, ‘What is known about the impact of professional entry medical education in rural places on rural medical practice location following graduation?’ guided the search strategy. Acknowledging graduate entry programmes, for students who already hold a degree, the term professional entry medical education was used to denote the first medical degree a person completes for entry to practise as a doctor.

### Identifying relevant studies

In designing scoping reviews, there is a need to establish key search terms to achieve a balance between a rigorous review and practicalities such as time and cost [38]. Here, we developed a Boolean string from key search terms and used truncated words and wildcards (in this instance *) to broaden the search and ensure all terms with the same root word were included. Search terms are in Table 1.

**Table 1:** **Key search terms used in the review**

**Population and course**	**Location and training**	**Time**	**Practice**
Medic* AND (Student OR Preregistration OR Undergraduate OR University)	(Rural* OR Regional OR Remote) AND (Preceptorship OR clinical placement OR Rotation OR clinical training)	Whole OR Part	Rural practice OR Actual practice OR Practice location

A preliminary search of Google Scholar showed the likely extent of findings and relevance of terms selected and helped to refine inclusion and exclusion criteria. The search results are not included due to the inability to accurately replicate Google Scholar searches [40]. Inclusion and exclusion criteria, consistent with our review purpose, were developed (see Table 2). The search concluded in September 2014.

**Table 2:** **Inclusion and exclusion criteria**

**Inclusion**	**Exclusion**
Peer reviewed articles published in English from any country	Articles not published in English and non-peer reviewed
Focus medical education	Other health professionals/cohort not defined
Published—post-1990	Published—pre-1990
Professional entry medical education	Postgraduate courses for qualified doctors
	Continuing professional education
	Continuing professional development
	Specialisation
Quantified amount of medical education in rural location	Amount of medical education in rural location could not be quantified

A search of the Cochrane Library identified no systematic reviews that were directly relevant to our research question. Databases searched included Medline, CINAHL, Proquest, Expanded Academic and Informit.

### Study selection

**Figure 1:**
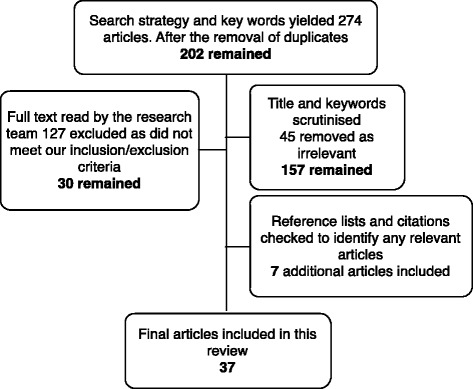
The process of article selection.

Using the developed search terms, 274 articles were identified, and after removal of duplicates, 202 remained. The title and keywords of the articles were compared with the inclusion and exclusion criteria, with the research team agreeing and confirming the elimination of irrelevant studies. Of the 157 articles that remained, the full-text versions of the articles were read by a minimum of three researchers. Through a process of matching against inclusion and exclusion criteria, 127 references were excluded. Primary reasons for exclusion were the following: literature review or commentary, the dependent variable was not rural practice, the authors did not quantify the time spent in rural areas or the authors considered intention rather than actual practice. The 30 identified articles that matched our inclusion and exclusion criteria were deemed directly relevant to our research question. We hand-searched reference lists from these articles and checked citations and identified seven further relevant articles. A total of 37 articles were included in our final review. To ensure that we had captured all articles, we completed an additional hand search of the reference lists of all articles that had been extracted for full-text review. No further articles were identified. The process of article selection is outlined in Figure 1.

### Data charting and collation

Consistent with the fourth stage of Arksey and O’Malley’s framework, the following is reported for each article: author, publication year, location, sample, method, major findings, discussion points and limitations. During data charting, a set of types of studies emerged, and we developed a typology (see Table 3) to assist with article sorting and structuring information for the reader.

**Table 3:** **The typology used in our review**

**Category**	**Typology**
1	Studies comparing rural work outcomes for rural versus urban campuses of the same university
2	Studies examining rural work destination of graduates of rural medical schools (i.e. medical education provided completely in a rural place)
3	Studies of the impact of partial rural medical education
4	Studies of impact of various durations of rural medical education on rural postgraduate internship
5	Studies that identify rural practitioners and investigate an association with rural medical education

The results presented in each typology category are detailed in Tables 4, 5, 6, 7 and 8. In charting the data, we were focused on consistent reporting. All available details are included in Tables 4, 5, 6, 7 and 8. Where detail is missing, the issue was not addressed in the study reviewed.

**Table 4:** **Typology category 1: studies comparing rural work outcomes for rural versus urban campuses of the same university**

**Authorship and location**	**Sample and method**	**Major findings**	**Discussion points/limitations**
Brokaw et al. [42] USA: Indiana University School of Medicine (IUSM)	2 487 IUSM graduates matched to American Medical Association Physician Masterfile to determine practice site. Students with first 2 years at eight regional campuses (*n* = 1 211) were compared with main city campus (*n* = 1 200). Multivariate logistic regression used.	Compared to city students, those who attended five of the regional campuses were more likely to practise in the region. Overall, attendance at any regional campus was a significant predictor of practice outside the city.	Regional campus students spent only first 2 years at regional campus.
Crump et al. [41] USA: University of Louisville School of Medicine, Kentucky	1 391 graduates (60 from rural Trover campus) matched to American Medical Association Masterfile to determine practice site. Compares students of rural Trover campus with main city campus students. Descriptive frequencies, percentages, means and standard deviations calculated.	Trover graduates were six times more likely to choose a non-metro area as a practice site (*p* = 0.001).	Students self-selected and were then interviewed. 68% of Trover students were from a rural hometown.

**Table 5:** **Typology category 2: studies examining rural work destination of graduates of rural medical schools (i.e. medical education provided completely in a rural place)**

**Authorship and location**	**Sample and method**	**Major findings**	**Discussion points/limitations**
Inoue et al. [43] Japan: Jichi Medical School (JMS)	1 871 JMS graduates. Postal survey.	‘Almost 100%’ response. 792 (42%) graduates working in rural prefectures.	Cannot compare with other Japanese medical schools as data not collected. Entrance exam focused on students intending to return to home prefecture.
Magnus and Tollan [44] Norway: Tromso School of Medicine Northern Norway	417 Tromso graduates. Postal survey.	84.2% response. 56.1% were working in ‘remote areas’/Northern Norway.	43.3% were reportedly raised in Northern Norway.
Sen Gupta et al. [32] Australia: James Cook University	530 JCU graduates. Longitudinal cohort study of graduates.	1–7 years following graduation. 59% worked rural at some point compared with 40% metro.	Approximately 14% had a bonded scholarship. Those with rural hometown and rural internship were most likely to work rural.
Stratton et al. [45] USA: University of North Dakota School of Medicine (UNDSM)	2230 UNDSM graduates postal survey.	41% response. 29.5% work in communities of 25 000 or fewer people.	Study focused on impact of curriculum expansion on retention of North Dakotan students in North Dakota practice following graduation.

**Table 6:** **Typology category 3: studies of the impact of partial rural medical education**

**Author and location**	**Sample and method**	**Major findings**	**Discussion points/limitations**
Forster et al. [61] Australia, University of New South Wales (UNSW)	*n* = 315. Retrospective online survey of graduates who spent 1–3 years of undergraduate training in UNSW Rural Clinical School.	214 respondents (68%); 26% are currently working in rural. Incremental effect of 1–3 years of rural exposure.	Self-reported on type (e.g. rural) of current work location.
Glasser et al. [51] USA: Illinois	*n* = 159. Effect of Rural Medical Education (RMED) Program and add-on curriculum of monthly sessions during the first 3 years of medical school and a capstone in the fourth year. Database on RMED students, graduation, location and specialty of residency.	Of the 103 grads in practice, 69 (67%) are in towns ≤20 000 or rural communities.	Does not compare with non-RMED or national data for rural practice. The RMED recruiter makes yearly trips to feeder schools to meet with sophomore, junior and senior students who have an interest in rural medicine.
Halaas [54] USA Minnesota	*n* = 1 063. Rural Physician Associate Program (RPAP) graduates. RPAP is a 36-week community-based elective placing students in rural communities of 3 000–20 000. Method not specified.	521 (62%) of RPAP students in practice at time of measurement were physicians in rural communities.	Does not specify where the data come from.
Halaas et al. [54] USA: Minnesota	*n* = 1 175. Rural RPAP graduates. Used RPAP database and descriptive statistics.	448/901 (49.7%) of currently practising graduates are in rural settings. 44% have practised in a rural setting all of the time. Proportion of RPAP graduates in rural settings is higher than 9% USA figure.	
Jamar et al. [59] Flinders University Australia	*n* = 124. Retrospective study of graduates who completed a rural fifth year.	Response rate 74 out of 124 (58.2%). Eight years after graduation, 20.8%–34.1% were located rurally; average of 21.8% per year over this time.	Voluntary programme—those interested in rural practice may have biased results. Does not compare percentage working rurally with a non-rural cohort or with a national figure.
Kane et al. [49] USA: University of Missouri School of Medicine	*n* = 168. Questionnaire of Summer Community Program graduates: second year medical students working with rural physician preceptors (4–8 weeks).	*n* = 78 (46%) were in rural practice for their first work location. Compared with non-participants, summer participants were more likely to work in rural locations for their first practice. 46% compared with 11% nationally that practise rurally.	Unable to compare participants with non-participants due to lack of full data set.
Lang et al. [50] USA: Dept. of Family Medicine at East Tennessee State University	*n* = 134. Effect of 4-week summer elective clinical preceptorships in southern Appalachia. Database of practice locations linked with databases from medical schools and other sites.	Of the 134 former students whose practice locations were identified, 44 (33%) are in rural areas compared with 9% of all physicians.	Small numbers over an 18-year period.
MacDowell et al. [52] USA: Illinois College of Medicine at Rockford	*n* = 160 RMED and 2 663 non-RMED graduates. Compared data on Rural Medical Education (RMED) Program and non-RMED students. RMED is add-on programme in years 1–4 plus 16 weeks in a rural practice in year 4.	56.3% RMED graduates are working in small towns or rural communities. RMED graduates reported more than 17.2 times more likely to be currently practising in a rural location (excluded those in residency), compared with all other U. Illinois medical graduates.	
Orzanco et al. [46] Canada: Universite de Sherbrooke (UdeS) and University of British Columbia (UBC) medical schools	*n* = 180 (UdeS), *n* = 194 (UBC). Linked students’ personal data and undergraduate MD programme to practice location data from the Canadian Post-MD Education Registry. Retrospective analysis with multiple regression analysis.	Significant difference in no. of weeks family practitioners practising in non-metro had spent in non-metro clerkships (*p* < 0.000 1). Median time 7.7 weeks compared with 3.9 weeks for those practising in other types of areas (UdeS). For UBC, none of those doing non-metro clerkship were likely to establish non-metro practice.	Length of clerkship in non-metro areas was the strongest predictor of location of practice for UdeS and ‘some’ relationship for UBC but small sample size. Noted lack of quality data.
Playford et al. [60] University of Western Australia	*n* = 1 017 (258 from rural clinical school; 79 urban) Cohort study comparing those in a rural clinical school (fifth-year rural placement) and those not.	Of 258 rural clinical school graduates, 42 (16.3%) were working rurally compared with 36 of 759 (4.7%) in the non-rural clinical school control group.	Voluntary programme—those interested in rural practice may have biased results. Rural background did not have an independent significant effect.
Quinn et al. [19] USA: University of Missouri School of Medicine	*n* = 48 rural programme graduates with *n* = 506 non-participants. MU-RTPP = summer community programme pre-second year, rural clerkship in third year and rural elective in fourth year. Tracked all participants of MU-RTPP, using database of postgraduate specialty training, practice locations and professional and public sources such as Board of Medical Specialties databases.	57.4% of graduates from MU-RTPP cohorts chose to practise rural or mixed rural county. Over 57% chose a rural location for their first practice. Compares 57.4% with 9% it states work in rural nationally.	Unable to make comparisons with non-participants because data incomplete.
Rabinowitz [55] USA Philadelphia Jefferson Medical College	*n* = 148. Data of current practice locations of graduates in the Physician Shortage Area Program (PSAP). PSAP required third-year clerkship at one of two non-metro locations.	PSAP graduates were around four times more likely than non-PSAP to practise in rural areas 39% *vs* 11%.	
Rabinowitz et al.* [56] USA Philadelphia Jefferson Medical College *Rabinowitz and Paynter (2000) [75] provides the same data but with a different focus.	*n* = 206. PSAP graduates. Retrospective cohort study 1978–1991. Using data from JMC Alumni Association to tracked PSAP and non PSAP graduates at Jefferson Medical College.	PSAP graduates were 32/150 (21%) of family physicians practising in rural Pennsylvania who graduated from one of the state’s seven medical schools although they are only 1% of graduates from those schools. 68 (34%) of PSAP grads were practising rurally anywhere in USA compared with 303 (11%) of non PSAP.	
Rabinowitz et al. [57] USA: Pennsylvania Jefferson Medical College	*n* = 38 PSAP graduates and 54 non-PSAP graduates, 11–16 years after graduation. Longitudinal follow-up of PSAP graduates.	After 11–16 years, 26/38 (68%) PSAP graduates were practising in the same rural area, compared with 25/54 (46%) non-PSAP (*p* = 0.03). Survival analysis showed PSAP graduates practise in the same rural locality for longer than non PSAP (*p* = 0.04).	
Smucny et al. [58] USA: New York State	*n* = 132. Rural Medical Education (RMED) Program graduates. RMED is 36-week clinical experience in rural communities in year 3. Physician masterfiles of American medical Association to compare practice locations for RMED with non-RMED used.	76 RMED graduates (58%) completed the questionnaire. 56/69 (81%) had completed postgraduate training. 26 % of RMED practised in rural areas (22/86), compared with non-RMED 95/1307 (7%).	59% RMED respondents considered their home town to be rural.
Strasser et al. [22] Australia: Monash University Bachelor of Medicine/Bachelor of Surgery Programme	Number of questionnaires distributed is not given. Retrospective cohort mail survey of four groups of students with different rural/urban background and experiences in rural medical education.	*n* = 243 responding higher mean in total number of weeks of rural placement was associated with a current practice location in a rural community rather than an urban community (*p* = 0.05), but not with first practice location (once vocationally qualified) (*p* = 0.16).	Author reports that rural/urban background had a significant interaction with all of the main outcomes except current place of practice.
Williamson et al. [19] New Zealand: Otago University Faculty of Medicine	*n* = 367; 293 after exclusions. fifth-year medical students from 2000–2001 were identified by the enrolment and alumni records. Study cohorts from three campuses (one of which has a 7-week undergraduate ‘rural health course’) were posted a questionnaire. Non-responders were followed up.	177 (63%) returned, of which 30 were ‘Gone, no address’, leaving 147 (50%). There was no significant difference among schools in the proportion of students working in rural areas.	Small numbers and 50% response. Content of the 7-week course is not described, although described as a ‘rural rotation’.

*Provides the same data but with a different focus.

**Table 7:** **Typology category 4: studies of impact of various durations of rural medical education and rural postgraduate internship**

**Author and location**	**Sample and method**	**Major findings**	**Discussion points/limitations**
Clark et al. [22], Australia, University of Sydney	*n* = 448 (98 had completed 32 weeks rural placement). Questionnaire on entry and exit from medical school.	8.1 % accepted rural internship. Those that completed 32 weeks rural placement were twice as likely to accept rural internship (21.3% *vs* 9.9 %).	Those undertaking the long rural placement did so because they already intended to go rural. Students undertaking extended rural placement were more than three times as likely as those with rural backgrounds to express preference for a rural internship. (23.9% *vs* 7.7%; *p* = 0.008).
Eley and Baker [63] Australia, University of Queensland	*n* = 28 completed questionnaire (in aggregate). Year 4 exit questionnaire of rural clinical school (RCS) programme and data on internship choice. Statistical tests compared answers to questions about experience of rural clinical school with internship choice.	Six out of 27 chose to return to their undergraduate placement hospital for internship.	No. of participants was too small to detect differences between sites. Lack of information about the hospital sites.
Eley and Baker [64] Australia, University of Queensland	*n* = 27 Undergraduates exiting RCS programme. Questionnaire and data on internship choices.	14/27 went to internships in large rural centres with 25 000-100 000 population in 2006.	
Eley et al. [65] Australia: University of Queensland (UQ)	*n* = 180. A retrospective web-based survey of all graduates who undertook clinical year 3, year 4 or years 3 and 4 at UQ rural clinical school 2002–2006.	124 replies (69% response). 29% working in rural places with population ≤100 000. Most important factor affecting rural workplace choice was spending 2, as opposed to 1, year at a rural clinical school.	69 % response rate so maybe selection bias possible in that rural workers respond to a rural focused research project. Only 7% of interns were in places with ≤25 000 population.
Eley et al. [65] Australia: Queensland	*n* = 631 internships. Analysis of number, source and location of interns by rural classification from university and health department records (2003–2008). Compared University of Queensland (UQ), UQ Rural Clinical School (UQRS) and James Cook University (JCU), which it implies is defined as equivalent to UQRS.	Rural clinical schools (JCU/UQRCS) were more likely to supply interns to hospitals in places with ≤100 000 than to major city hospitals.(OR, 8.8; 95% CI, 4.6–16.7; *p* < 0.000 1; OR, 6.5; 95% CI, 3.5–12.2; *p* < 0.000 1)	Study focus is on producing interns for Queensland from Queensland Universities.
McDonnel Smedts and Lowe [67] Australia: Flinders University Northern Territory (NT).	*n* = 452. Statistical analysis of de-identified data from a database of place of internship and medical registration of Flinders medical graduates 1999–2005.	Those doing final year in at NT clinical school were more than 10 times more likely to complete their residency in NT (54 % did so).	If students were from NT and attended NT clinical school, 70% completed residency in NT.
McDonnel Smedts and Lowe [68] Australia: Northern Territory Clinical School of Flinders University	*n* = 683. Retrospective analysis of medical school and hospital data on all medical students who completed a placement with the Northern Territory Clinical School (NTCS) between 1998 and 2007.	Placement length was a significant predictor of an NT internship (*p* < 0.05; OR, 1.08; 95% CI, 1.07–1.09).	Did not identify whether medical students had a rural background.
Playford and Cheong (2012) [27] Australia: University of Western Australia	*n* = 490. Undergraduate data were linked with postgraduate placements to provide dataset on rural exposure history of junior medical practitioners.	Participation in a longer rural placement at RCSWA was associated with significantly more postgraduate year 1 rural work compared with a short placement alone (OR = 1.5, CI 0.97–2.38).	Interns are classified as working in rural if they do 4 weeks in rural out of the year. This is a very short amount of time to classify as rural. Rural-origin practitioners were more likely to take rural rotations in postgraduate years.
Sen Gupta et al. [66] Australia: James Cook University (JCU) Queensland and other Australian medical schools.	*n* = 292. Exit survey of JCU graduates compared with Medical Students Outcomes Database data for eight other, largely metropolitan, schools.	67% of JCU graduates undertook their internship outside a metropolitan centre compared with 17% of others (OR: 10.0), and 47% in outer regional centres compared with 5% of others (OR: 16.6).	46% of JCU graduates intend to practise in outer-regional, remote or very remote areas, compared with 15% for other Australian universities.

**Table 8:** **Typology category 5: studies that identify rural practitioners and investigate an association with rural medical education**

**Author and location**	**Sample and method**	**Major findings**	**Discussion points/limitations**
Pathman et al. [71] USA	*n* = 456. Two postal surveys of primary care physicians who had moved to rural practices 1991 and 1996–1997. Considered where and when attended medical school and number of months in rural as a medical student.	456 responded to both surveys (69%). More than half of those working rurally had participated in rural rotations as students.	Included only those who were working in rural areas.
Rourke et al. [70] Canada	507 rural family practitioners in Ontario Medical Association, compared with 505 randomly selected from practising in places with population >50 000. Postal survey.	Response of 484 (47.8%); 264 rural, 179 urban. Rural were 1.8× more likely to have spent ≥8 weeks in a rural setting during undergraduate medical training compared with urban.	
Rosenblatt et al. [72] USA	1 991 practice locations of USA medical graduates 1976–1985. Practice location determined using American Medical Association masterfile, includes year and place of medical school and current practice location.	12.6% were practising in rural areas. Much variation between medical schools: University of North Dakota highest (41.2%). 12 medical schools produced over 25% of graduates working rurally. Strongest association was between % of graduates working rurally and rurality of state where medical school is located.	Study focused on all medical graduates and then clustered programmes by rural state. No specific focus on location of education within the states.
Rolfe et al. [73] Australia: University of Newcastle Medical School	*n* = 217. Linked graduate data from a survey with Faculty of Medicine admissions database. Cross-sectional survey of University of Newcastle medical graduates.	226 (68.3%) response. After exclusions 162/217 (75%). 22% of post-interns working in rural. Those who chose a rural location for the general practice attachment were 3.02 (95% CI: 1.25–7.32) times more likely to be working in a rural area than those who chose an urban location.	Limitation students chose year 5 attachment. There was a significant relationship between a rural background and currently practising there.

## Findings

Consistent with Arksey and O’Malley’s [39] framework, findings and reporting provide an overview of the articles selected for review. As the purpose of the scoping review was to identify what is known about an association between professional entry medical education in rural places and rural medical practice location following graduation, reporting focuses on the types (often related to durations) of rural medical education, the effect of this education on employment location following graduation, any key messages and limitations about the link between rural medical education and rural employment, as highlighted by study authors. We were interested in whether studies included discussion of cost-effectiveness, but following a review of all, we found none that considered this issue.

### Typology category 1: rural versus urban campuses of the same university

In Table 4, we summarise studies that compare rural practice location outcomes for rural versus urban campuses of the same university.

Findings from the two studies reviewed [41,42] indicated that those who attended rural campuses are more likely to practise in non-metropolitan areas than those who attend the city campus of the same university. There are limitations to these findings, associated with student self-selection, with the hypothesis that students who choose to study in rural campuses have a predisposition to work outside main cities; for example, in the article by Crump et al. [41], 68% of students who attended the Trover campus came from a rural hometown.

### Typology category 2: graduates of rural medical schools—medical education provided completely in a rural place

Table 5 summarises four studies where researchers considered the effect of all medical education located in a rural place on the production of rural doctors [32,43-45].

These articles demonstrate the diversity of ways that rural is depicted. Jichi Medical School [43] was described as located in a country town with a population of less than 20 000 approximately 100 km north of Tokyo. The Tromso medical school in Northern Norway [44] was described as 70° north of the Arctic Circle. In the article by Stratton et al. [45], North Dakota, USA, is described as ‘*a rural state*’.

Collectively, study findings indicate that, variously, around 30%–56% of graduates at the time of the studies were working in rural places: Jichi 42% [43], Tromso 56.1% [44] and North Dakota 29.5% [45]. Sen Gupta et al.’s [32] study showed that 59% of James Cook University medical graduates had worked in non-city areas at some time during the first 7 years postgraduation. The researchers in each article note that the medical schools were developed specifically to address rural workforce challenges, to address the workforce needs of local ‘underserved populations’ [32] and to ensure graduates for rural areas [43-45].

For Jichi [43] and Tromso [44], findings were not compared with rural doctor outcomes for other medical schools in the same country due to unavailable data. As with category 1 of our typology, success in delivering rural doctors could be attributable, to some extent, to students’ predisposition to work in rural areas. Differences in the percentage of graduates working in rural areas between Tromso at 56.1% [44] compared with North Dakota at 29.5% [45] could be linked to the measurement of different outcomes. For North Dakota, the statistics relate to working in communities with up to 25 000 residents, while for Tromso [44], it is Northern (less populated) as opposed to Southern (more populous) Norway. There are differences in programme focus. As an example, Stratton et al. [45] described the focus of their study as retention of North Dakotans in North Dakota, while Inoue et al. [43] were concerned with doctors working in rural prefectures.

### Typology category 3: studies of the impact of partial rural medical education on rural medical recruitment

In Table 6, we summarise studies that consider the impact of medical education partially in a rural area on producing rural doctors.

These studies [19,46-61] consist of two main types: evaluations of the effects of special rural programmes (all from the USA) and evaluations of varying lengths of location in a rural area for GP attachments or attendance at ‘rural clinical school’. Fourteen of the 17 studies support positive effects of partially rural medical education on the production of rural doctors. Three studies (two Australian, one Canadian) indicate incremental effects of increasing time spent in rural places for medical education [46,47,61], although Orzanco et al. [46] only found this for one of the two universities included; for the other, findings were inconclusive. In New Zealand, Williamson et al. [48] did not find an association between rural placement and rural working. For the 11 studies of USA rural programmes, percentages of graduates who proceeded to work in rural places range from 26% [58] to 67% [51].

Some of the USA studies compare the effects of rural programme graduates versus non-programme graduates. Examples include Smucny et al. [58] (26% of graduates from a rural programme working in rural areas compared to 7% non-programme). Rabinowitz et al. [56] found that, after 11–16 years, 68% of rural programme graduates worked in rural areas, compared to 46% for non-programme graduates. Most USA rural programme studies compare the impact of rural programmes with a figure for aggregated national medical schools, variously stated as producing between 9% and 11% of all USA medical school graduates that work in rural areas.

Many of the USA programme studies described small numbers of graduates produced; for example, Lang et al. [50] report 134 graduates over 18 years, with Rabinowitz [55] reporting on a total dataset of 148 graduates. Several of the USA studies are of the same programme over time. In total, 16 of the 17 studies consider the output of one university only. The exception is Orzanco et al. [46] who include findings from two Canadian universities.

As previously noted, positive findings about graduates working rurally may be confounded by students’ rural origin or choice to undertake rural programmes, attachments or years at a rural clinical school. Glasser et al. [51], for example, note that their recruiters visit rural secondary schools to identify those interested in rural medicine. Anomalously, Strasser et al. [47] conclude that rural/urban background had a significant interaction with all their main outcome measures except place of current practice.

### Typology category 4: studies of impact of various durations of rural medical education and rural postgraduate internship

We found nine studies [27,62-69] where the impact of various durations of rural medical education on rural employment immediately following graduation (i.e. for postgraduate internship) was considered. These are outlined in Table 7:

In two studies, small numbers of annual graduates from the University of Queensland were considered [63,64]. Those undertaking a rural placement, compared with no rural placement, or a long rural placement compared to a short rural placement, were more likely to undertake a rural internship [27,62,65,69]. Some studies identified whether students were from a rural place or intended to work rurally and noted that this had a positive impact on eventual rural internship [67]. Clark et al. [62] found that extended rural placement had a stronger association with rural internship than coming from a rural place. A high rate of rural internship outcome was shown for James Cook University (rural Queensland, Australia), with 67% of graduates undertaking their internship outside a metropolitan centre. This compares with 17% from other Australian universities [66]. Diverse ways of defining rural were again evident; perhaps the most challenging being Playford and Cheong’s [27] designation of rural internship as having at least 4 weeks in 1 year in a rural place.

### Typology category 5: studies that identify rural practitioners and investigate an association with rural medical education

The final category in our typology is presented in Table 8.

We found four studies in this category [70-73]. Rourke et al. [70] and Pathman et al. [71] examined associations between working in rural areas and amount of time spent in rural rotations as students, showing some associations. They did not consider gradient effects. Rolfe et al. [73] found a positive association between rural working and having a rural general practice placement attachment. Findings of Rosenblatt et al. [72] indicated that a small number of USA medical schools have much higher production of rural doctors, but they did not explore for rural location of the medical schools or duration at a rural location during medical education.

## Discussion

The purpose of this review was to scope the state of evidence in relation to two research questions: Does medical education in a rural place produce rural doctors? And, is there a gradient of time spent in rural-based medical education that contributes to the increased likelihood of a graduate working in rural medical practice? We found few studies that specifically sought to identify the benefits of having medical education wholly in a rural place, or of gradient effects of increasing time spent in rural medical education, on producing rural doctors. Studies found were limited by considering outputs of single medical schools, lack of comparative data and/or being conducted by researchers from the medical school being studied. We found no studies considering the cost-effectiveness of different models of producing doctors for rural places. While most of the papers reviewed showed evidence that rural exposure during medical education increased the likelihood of practising rurally following graduation, insufficient detail, differing definitions, small numbers, inclusion of outputs from only one university, confusing reporting and lack of comparative data make drawing clear conclusions about both of our research questions unwise and impractical. The best we can say is indications are positive, but better quality evidence is needed. Our reporting is crude because we endeavoured to unite findings to obtain a sense of the overall state of the evidence.

Category 1 of our typology (two studies found) compared rural campuses with the main campus of the same university and appeared to show that rural campuses produce higher percentages of rural workers. Typology category 2 examined studies of the impact of medical schools providing education wholly in rural places. From the studies, we conclude that percentages working rurally appear high when compared with available national data. Typology category 3 would ideally have provided evidence about gradient effects as it unites 17 studies each defining lengths of time medical students spent in rural places during their undergraduate degrees. Three studies considered different lengths of time spent in rural education, concluding an association between longer time in a rural clinical school or GP attachment and greater likelihood of rural working. Most included studies showed associations between time spent in rural programmes, clinical schools or attachments and rural work outcomes. Typology category 4 considered first years of employment in internships. Again, evidence indicates a relationship between experiences of medical education in a rural place and rural working. The most striking finding is from Sen Gupta et al. [66] of around two thirds of students proceeding to a rural internship following wholly rural education. Typology category 5 considered studies involving rural doctors and identifying factors associated with rural working. Again, there were indications, in most included studies, of links between time spent in rural medical education and rural work outcomes.

Overall, we found some studies confusingly written, particularly around identifying numbers of students or practitioners included in analyses relating to our research question. Considering the impact of place was often not the papers’ focus but was explored alongside other variables and relationships. Some studies had very small sample sizes. Some lacked transparency, for example, by not reporting original sample size or methods. Several studies had to be excluded from our review, as they did not quantify precise periods of rural exposure. Some studies combined (and in some cases conflated) intentions to work rurally with actual rural work. With studies considering programmes, GP attachments or time spent at rural clinical schools, it might be argued that what is being measured is not simply the effect of rural education location *per se* but, rather, the ‘package’ of experience provided, including ‘hard to measure’ factors like the enthusiasm of educators and mentors for rural working. None of the associations linking place of education with place of employment are therefore independent of other factors. Invariably, the studies of medical school students’ conversion to rural practice, following the range of rural education durations and types, were conducted by teams involving researchers from the medical schools concerned.

There was considerable variety in the ways rural was discussed and defined. This is a regular challenge when comparing studies in rural health. We included studies where authors specifically stated they were considering rural working as an outcome; that is, they specifically used the term rural (remote and regional were also included), and they specifically defined or described what was meant. For example, Inoue et al. [43] note there is no definition of rural in Japan and state: ‘In this study, rural areas were defined as areas that are subject to certain laws in local municipalities: the Special Promotion Act for Sparsely Populated areas, the Mountain Village Promotion Act, or the Special Act for Areas with Heavy Winter Snow’. Australian studies tended to use established categorisations, for example, the Rural, Remote and Metropolitan Areas Classification (RRAMA) [74]. USA studies used various categorisations, including ‘rural–urban commuting area (RUCA) codes’ and Rural Urban Density Typology, categorising variously towns and counties on a continuum of rural to urban. Other studies applied population size of towns/cities or population size plus distance from a large centre as proxy for rural; for example, Rourke [70] defined rural communities as having populations of 1 000–30 000. Forster et al. [61] used participant-defined location—and was the only study reported here to do so.

Given the caveats outlined, there is evidence (lacking in robustness though it is) that does suggest that medical education in a rural place may positively affect production of rural doctors. Additionally, there is some evidence to suggest a gradient effect, with increasing duration of educational experiences in rural settings, although the number of studies is small and limited to evaluating single programmes.

A key challenge to having these findings acknowledged as interesting and worthy of further exploration is the regular reporting that the findings are likely confounded by students’ having a rural background or pre-existing propensity to choose rural work. This is often suggested to influence their initial choice of a rural-based medical programme, special programme or attendance at a rural clinical school.

While rural background is influential, its inclusion as a problematical confounding factor, detrimental to considering the value of rural medical education, raises questions. The success of rural medical schools such as Jichi, Tromso and James Cook University in producing high proportions of rural doctors prompts consideration of a counterfactual. Put simply, what happened to these rural-origin students before the rural programmes existed? Did the students attend city universities and become converted to urban work? Or, did they not attend medical school at all? Are rural medical programmes, therefore, simply efficiently finding those with a propensity to rural work and converting them into rural doctors? It is an intriguing, but inherently un-researchable, question to compare the situation now with the past.

### Limitations and recommendations

Our interest in conducting this review was focused on exploring an association between rural medical education and rural working. We were specifically interested in rural practice following medical school, so we did not include the impact of postgraduate study. A review that considers studies reporting work location following the completion of all postgraduate education would have a different purpose to ours.

Confounding factors feature in discussions considering the relationship between rural medical education and rural practice location—most notably the effect of rural background. Due to varying reporting in the studies we reviewed, we made the early decision not to divide studies into those that stated they had controlled for rural background and those that did not state this. Put simply, we did not have the consistent information for every study about whether or not they had controlled for rural background, so it would be misleading of us to attempt to divide. Of the few studies that stated they had accounted for rural background, the strongest predictors for practising rurally, following graduation, were location of the medical school and time spent gaining experience in rural practices during medical education. There is a gap in robust studies which allow for distinction of the effects of different variables on rural practice outcomes.

We believe that the reporting typology that we developed provides a useful, if pragmatic, structure for reporting about a relatively large group of articles. Providing an overview of studies from different countries, and typologising them, given variable coverage in reporting is inevitably challenging. Consistent with scoping review methods, the purpose was to map the nature and extent of existing international published evidence.

The WHO [6] recommends medical education in a rural place as one of a basket of means to increase rural doctor supply, while—to date—suggesting that the evidence base can be merely indicative (due to study paucity and methods). Within the evidence gap, traditional city-based medical schools continue to provide small-scale self-evaluations of their rural-focused programmes. In the studies that we reviewed, there was a lack of description of independent, or external, scrutiny in evaluation and auditing of data. There is a need for studies incorporating these strategies.

For countries that lack access to well-constructed longitudinal medical workforce databases, there is a dearth of quality comparative statistics. International collaborations that collect comparative data would strengthen the evidence base. Findings from independent, international, robust, studies would be valuable because rural advocacy groups continue to state rural doctor deficits, while city-based medical schools continue to provide small-scale evidence that their programmes are working.

Given the considerable public finance invested in schemes to generate rural doctors, including bonded student places, clinical schools, workforce agencies and work incentives [6], independent studies that consider the relative cost-effectiveness of rural-based medical education versus other strategies would be useful for policymakers.

Studies to address the question—does medical education in a rural area most effectively produce rural doctors?—might adopt modelling or prospective or *post hoc* approaches. Ideally, a study would include the following: rural and urban medical schools; apply a practical, but meaningful, classification of rural; include data on students’ rural/urban background on entering medical school (to establish the effects of rural origin *vis a vis* rural medical education); apply a measure of rural work outcome (e.g. place of work after 5 years or durations of work in rural/urban places); and identify *per capita* costs of producing a medical graduate in a rural or urban area.

A study to establish whether there is a gradient in rural work outcomes in relation to time spent in rural places during medical education would ideally include the following: several rural- and urban-based medical schools; use data on students’ rural/urban background on entering medical school; identify number of weeks spent located in a rural place, as part of entry-level medical education for each student; apply a measure of rural work outcome; and identify costs associated with different numbers of weeks in rural places.

While such studies might be dismissed as unfeasibly complex or large and thus expensive, the ongoing cost, internationally, of medical workforce incentives and payments to universities to produce rural practitioners is considerable. The question is perhaps whether enough of the powerful stakeholders want to know the answers.

## Conclusion

Technology should make education of all types more readily available in future, regardless of location, but there will always be a strong element of medical education that involves hands-on work with patients and learning from skilled role models. The location where these experiences take place may be important, and it seems only just that rural populations, national governments and international agencies should have the best evidence so they can fund the best methods of producing rural doctors. Some of the significant international spending applied to getting doctors to work rurally could be diverted to finding out what works. In the short term, an outlay of resources to identify what works best could lead to considerable long-term gains in healthcare accessibility and ultimately to a cost-effective contribution to improving rural health outcomes.
